# Treatment expectations in glaucoma: what matters most to patients?

**DOI:** 10.1038/s41433-023-02532-w

**Published:** 2023-04-24

**Authors:** Atika Safitri, Evgenia Konstantakopoulou, Kuang Hu, Gus Gazzard

**Affiliations:** 1 Institute of Ophthalmology, University College London, London, UK; 2 NIHR Biomedical Research Centre at Moorfields Eye Hospital NHS Foundation Trust, London, UK; 3 Division of Optics and Optometry, University of West Attica, Athens, Greece

**Keywords:** Outcomes research, Glaucoma

## Abstract

**Background/Objectives:**

Recent clinical trials in glaucoma have used patient-reported outcome measures (PROMs) of health-related quality of life to evaluate interventions. However, existing PROMs may not be sufficiently sensitive to capture changes in health status. This study aims to determine what really matters to patients by directly exploring their treatment expectations and preferences.

**Subjects/Methods:**

We conducted a qualitative study using one-to-one semi-structured interviews to elicit patients’ preferences. Participants were recruited from two NHS clinics serving urban, suburban and rural populations in the UK. To be relevant across glaucoma patients under NHS care, participants were sampled to include a full range of demographic profiles, disease severities and treatment histories. Interview transcripts were evaluated using thematic analysis until no new themes emerged (saturation). Saturation was established when 25 participants with ocular hypertension, mild, moderate and advanced glaucoma had been interviewed.

**Results:**

Themes identified were: Patients’ experiences of living with glaucoma, patients’ experiences of having glaucoma treatment, most important outcomes to patients, and COVID-related concerns. Participants specifically expressed their most important concerns, which were (i) disease-related outcomes (intraocular pressure control, maintaining vision, and being independent); and (ii) treatment-related outcomes (treatment that does not change, drop-freedom, and one-time treatment). Both disease-related and treatment-related experiences were covered prominently in interviews with patients across the spectrum of glaucoma severity.

**Conclusions:**

Outcomes related both to the disease and its treatment are important to patients with different severities of glaucoma. To accurately evaluate quality of life in glaucoma, PROMs may need to assess both disease-related and treatment-related outcomes.

## Introduction

Glaucoma is a progressive disease characterized by damage to the optic nerve and visual loss which, if left untreated, can lead to blindness [1–3]. Glaucoma may affect activities of daily living such as reading [4], walking [5], or driving [6]. Falls and inability to work are also reported [7, 8]. Approximately 10% of 70 million people with glaucoma worldwide are bilaterally blind [9].

While the aim of treatment is to prevent loss of vision and thereby preserve health-related quality of life (QoL), it remains unclear how best to measure the success of treatment. Both generic and vision-specific patient-reported outcome measures (PROMs) have not demonstrated clinically meaningful differences in QoL in recent well-designed randomized controlled trials [10, 11]. It has been proposed that existing PROMs are not sensitive enough to function as primary end points [12, 13].

Current measures of QoL tend to emphasise the effects on the patient of the disease itself [14, 15]. However, glaucoma is a largely asymptomatic disease in its early stages, so available PROMs instruments may not be able to measure what they are intended to. Therefore, it is necessary to re-evaluate what really matters to patients who are living with glaucoma.

Few studies have assessed outcome preferences among patients with glaucoma. Kulkarni et al. used focus groups to evaluate preferences among patients using intraocular pressure (IOP) lowering eye drops and patients who had undergone glaucoma surgery [16] and reported patients’ ability to maintain independent living as the most important outcome. Le et al. [17] explored perspectives among patients with newly diagnosed ocular hypertension (OHT) or mild-moderate glaucoma who might be considered for minimally invasive glaucoma surgery. Disease-related outcomes (such as the ability to drive and maintain mobility outside home) and treatment burden were expressed as important, but their relative importance was not explored. A similar but extended set of preferences was subsequently reported for surgery-naïve patients with moderate to severe glaucoma [18]. Evidence is emerging that treatment for glaucoma may generate a significant burden for patients that may negatively impact QoL, separate from the burden of disease [19].

The aim of this study is to explore outcome preferences and treatment expectations of patients across a broad range of glaucoma severity and treatment history. This information needs to be elicited from patients themselves using a robust qualitative research design. In this study, we specifically ask patients to identify those outcomes that are of utmost importance to them.

## Subjects and methods

The study was designed and reported according to the Consolidated Criteria for Reporting Qualitative Research [20].

### Participant recruitment and ethical approval

Patients attending glaucoma outpatient clinics at two locations in the UK, namely Moorfields Eye Hospital NHS Foundation Trust and Croydon University Hospital, were approached to participate face-to-face by the interviewer (AS). One clinic is in an urban location (central London) and the other serves a mixed urban, suburban and rural population. No relationships with participants had been established prior to study commencement. Participants had to be diagnosed with open angle glaucoma (OAG, including primary open angle glaucoma, normal-tension glaucoma and pseudoexfoliative glaucoma) or OHT and to require or have had treatment to lower IOP. Diagnosis was made by a glaucoma specialist clinician. Patients with other ophthalmic pathology such as visually significant cataract were excluded. Participants were required to be able to understand, read and speak English without translation.

**Fig. 1:**
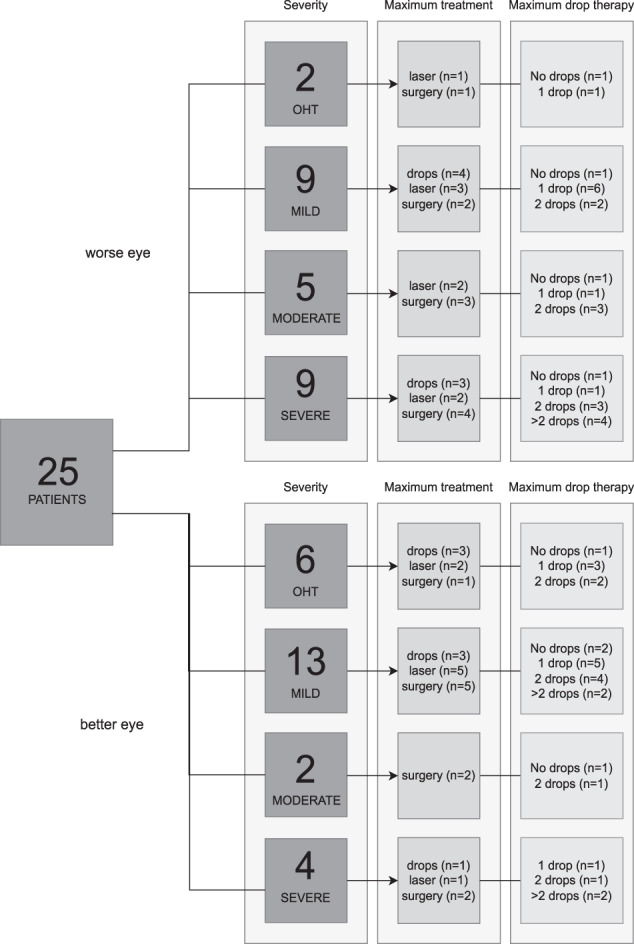
Purposive sampling covered a broad range of disease severities and treatment histories. Numbers of participants with corresponding disease severity and treatment history are shown. Worse eye and better eye were defined for each participant using Mean Deviation from Humphrey 24-2 visual field tests. OHT ocular hypertension.

Purposive sampling was used, where the participants were invited to participate based on the severity of their condition and their treatment history (Fig. 1). Specifically, we recruited participants covering a broad range of disease severity and treatment history. Ocular hypertension and early glaucoma are part of the same spectrum of disease. Both are treated and therefore it was both relevant and important to include participants with ocular hypertension in our study. Disease severity was classified for better and worse eyes separately using Hodapp-Parrish-Anderson criteria for Mean Deviation (MD) from Humphrey 24-2 visual field tests (Carl Zeiss Meditec, Dublin, CA, USA) [21, 22]. Additionally, we performed integrated visual field analyses to classify disease severity based on binocular visual function [23]. Treatment was categorized based on the maximum treatment experienced, defined by invasiveness: drops were considered to be the least invasive treatment and glaucoma surgery of any kind was considered to be the most invasive. The number of drops used was defined as the number of prescribed topical glaucoma medications.

Sample size was not predetermined, and no minimum sample size was stipulated. Our study aims to generate data on the diversity of patient expectations. Therefore, participants were recruited and interviewed iteratively until no new concepts emerged from successive interviews (that is, data saturation was achieved) [24]. Thematic analysis was conducted on interview transcripts shortly after each interview was conducted.

In total, 25 patients were approached and agreed to participate. The refusal rate was 0%. Participant characteristics are described in Table 1.

**Table 1:** Characteristics of participants.

Participant characteristics	
Characteristics	*N* = 25
Gender	
Female	10
Male	15
Age (years)	
Mean	67.7
Range	32–89
Ethnicity	
Caucasian	13
Asian	4
Black	8
Best corrected visual acuity – better eye (LogMAR)	
Mean (Standard Deviation)	0.2 (0.2)
Range	−0.08 to 1.00
Best corrected visual acuity – worse eye (LogMAR)	
Mean (Standard Deviation)	0.3 (0.45)
Range	−0.08 to 1.78
Diagnosis – better eye	
OHT	6
Mild OAG^a^	13
Moderate OAG^a^	2
Severe OAG^a^	4
Diagnosis – worse eye	
OHT	2
Mild OAG^a^	9
Moderate OAG^a^	5
Severe OAG^a^	9
Visual Field Mean Deviation (dB)	
Range – better eye	−32.12 to 1.53
Range – worse eye	−35.91 to −0.36
Range – integrated visual field	−31.19 to 0.79
Maximum glaucoma treatment experienced by patient	
Drops	7
Laser^b^	8
Surgery^c^	10
Maximum number of drops used by patient	
0	4
1	9
2	8
>2	4

*OHT* ocular hypertension, *OAG* open-angle glaucoma.

^a^Classification based on Hodapp-Parrish-Anderson criteria for Mean Deviation.

^b^Includes Selective Laser Trabeculoplasty.

^c^Includes penetrating surgery (aqueous shunt surgery, trabeculectomy), non-penetrating surgery, and minimally invasive glaucoma surgery.

The study was approved by the Northwest – Haydock National Health Service (NHS) Research Ethics Committee (20/NW/0347) and was conducted according to the tenets of the Declaration of Helsinki. Written informed consent was obtained from each participant prior to participation.

### Interview guide

**Fig. 2:**
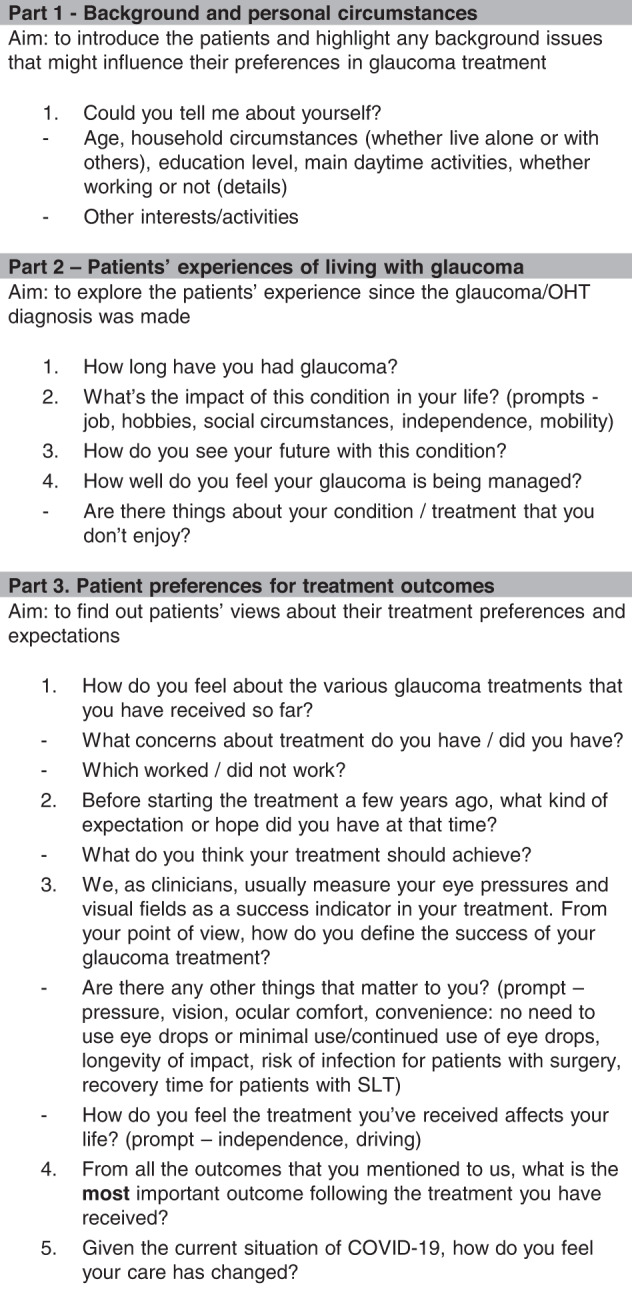
Topic guide. Used for semi-structured interviews.

The interview guide (Fig. 2) was adapted according to our research question from that used in a recent focus group study [16]. We specifically asked participants which outcome mattered most.

### Interview procedure

Semi-structured interviews were conducted and audio-recorded between November 2020 and February 2021. Interviews were carried out after the participants’ clinical consultations, in a private room within the hospital. Some participants chose to have a family member present in the room.

Interviews were conducted face-to-face in the English language by a female PhD researcher with a background in medicine and trained in qualitative research (AS). The interview process took between 11 and 42 min and the median duration was 24 min. Prompts were used to encourage participants to expand on a point, or to clarify a question where it appeared that the participant may have misunderstood the interviewer. Field notes were made during interviews to facilitate subsequent analysis. Repeat interviews were not undertaken.

### Analysis

Audio-recordings were transcribed verbatim by a professional transcription service. All transcripts were audited for accuracy, and they were not returned to participants. Transcribed data were analysed by one of the authors (AS) using thematic analysis. Themes were defined based on the study aims. The researcher grouped the data into themes and examined all the cases in the study ensuring all manifestations of each theme had been accounted for and compared. Responses to the question about the most important outcome were coded separately to eliminate bias in the analysis of responses. Nodes with more coding references than others were identified as prominent themes.

A second researcher (KH) independently read and analysed a subset of the transcripts. Discussion was conducted between the two analysts to avoid subjective judgements (reflexivity) and to solve potential limitations of different interpretation (triangulation). The second researcher also reviewed any transcripts for which the first analyst was unsure about classification. Full agreement on themes and coding was reached between the two analysts through these processes. Participant checking was not performed. The qualitative software package NVIVO 12 (QSR International, Cambridge, Massachusetts, USA) was used for data management.

Clinical information including disease severity and treatment history was extracted from patients’ medical records.

## Results

We identified key domains related to OAG and OHT, relevant to patients. Data were coded and developed into 4 key themes: ‘patients’ experiences of living with glaucoma’; ‘patients’ experiences of having glaucoma treatment’; ‘most important outcomes to patients’; and ‘COVID-related concerns’. Representative direct quotations taken from interview transcripts are presented for these themes and annotated with participant codes (Table 2). Subthemes that emerged from the analysis are also presented.

**Table 2:** Illustrative quotations categorized by theme and subtheme.

**Theme 1. Patients’ experiences of living with glaucoma**	
Activity limitation	*I feel like I’m more likely to make silly mistakes when I’m walking… I might not observe a step in the pavement. L014 (Severe OAG)*
Reading	*I want to be able to just, well read and go out and about, just lead a normal life like everybody does without any problem. L023 (Severe OAG)*
Peripheral vision loss	*I found out that my peripheral vision was not sharp enough for me as a civil engineering, I drive fast things and that sort of things. And I decide that my peripheral vision was not good enough for driving, so I don’t drive anymore. L017 (Severe OAG)*
Symptoms associated with glaucoma	*I can see the colours and everything but if I do just use this eye. I can see the yellow alright, but the letters aren’t very clear. It’s very fuzzy. L004 (Early OAG)*
Being independent	*Now I have to rely on other people, whereas normally I’d say to somebody, oh don’t worry, I’ll come along and pick you up and take you. L023 (Severe OAG)*
Psychological consequences	*I think… I feel like sometimes it might give me less confidence in… in some physical activity. L014 (Severe OAG)*
Disease stability or remission	*What matters the most is… is stopping… any further progression of the loss of vision in the right eye. L004 (Moderate OAG)*
Fear of blindness	*So, I want to have my sight until… I don’t want to lose my sight. That’s all I want. L012 (Severe OAG)*
Understanding the nature of glaucoma	*But the thing about something like the Glaucoma or the Ocular Hypertension… is you don’t know that you’ve got it. L013 (Early OAG)*
Acceptance of disease	*I wasn’t sure for the first couple of years I’ve had glaucoma treatment I didn’t know if I actually had glaucoma or if it was a mistake. L004 (Early OAG)*
Comorbidities or other health concerns	*I’ve been told that blood pressure affects your eyes and with glaucoma. L003 (Moderate OAG)*
Deterioration	*I can certainly tell it’s a lot worse than the left eye… just be doing that comparison with putting a hand over your face…over one eye and then the other eye. And that’s quite noticeable now. I’m sure it’s more noticeable than it was… two… three years ago. L002 (Early OAG)*
Vision improvement	*I was hoping when I first had the medication, thinking that the left eye would be like the right eye, but it doesn’t work that way because my left eye is worse than the right eye. L016 (Severe OAG)*
**Theme 2. Patients’ experiences of having glaucoma treatment**	
Activity limitation due to treatment	*If I’m going on a long-distance journey… then I don’t take the drops in the morning. Because I know my eyes will be… blur… they’ll be blurry. So, either I miss them for one day… or whatever. Short journeys doesn’t make any difference. L013 (Early OAG)*
Complying with treatment	*Sometimes you could be in a meeting, and you don’t have time for the eye drops. It’s only in the last few years I would take them to my work and put it on my desk so they’re visible to me and if I walked away from my desk when I come back, I’m like, oh my God I had better take it…so I think it was very haphazard. L011 (Moderate OAG)*
Symptoms associated with treatment	*But the second drop, I think it stings a bit, you know, it pains you, you know, when you put it in, it’s like sharp, it takes time to … to cool down. L022 (Early OAG)*
Problems with surgery	*I felt the pain, I felt the needle going in and I kept raising my hand and the anaesthetist and the surgeon were saying that they did their best. For some reason I could still feel, so I came off the, the, erm, the surgery and I was in tears because I could still feel that pain and, and they couldn’t understand. L011 (Moderate OAG)*
Problems after surgery	*That’s been quite disruptive because I’ve had so many surgeries. So, I’ve had quite a lot of periods where it’s been difficult to concentrate on work or I’ve had… I’ve had a lot of appointments here. L014 (Severe OAG)*
Multiple surgeries	*So, I’ve had two revisions I think… to the… to the stent thing. Yeah, so obviously no-one wants to be going in for like five surgeries… L014 (Severe OAG)*
Recovery from surgery	*It’s similar to um, well at the beginning it was like constantly in my eye, it’s watery and then that’s what they did, okay, we’ll decrease the er, we’ll decrease the er, Dexamethasone, and increase them two hourly, what the heck is going on? Two hourly, I said, excuse me. L015 (Moderate OAG)*
Problems with laser treatment	*I’ve taken the laser, I don’t enjoy the procedure. Mmm, I don’t, it doesn’t feel quick. So it’s the, so for me personally the first part of it is to take the drops in your eyes and it, the feeling that I get makes me feel a little bit nauseous and then because is suffer from migraines the bright lights. L019 (Early OAG)*
Problems after laser treatment	*Afterwards it feels like you’ve been punched in the eye a good couple of times before your eyes start to feel back to normal. L019 (Early OAG)*
Side effects of glaucoma drops	*It does make the eyes a bit, er, red and also, your eye, eyelids and the hairs there they, they change, you know, they, they turn and sort of point different directions, you know, it does affect the eyelids a bit, they become a bit sore. L007 (OHT)*
Understanding of glaucoma treatment	*it’s very easy to say just take the drops, take this, take that, but you don’t actually understand what it is. L011 (Moderate OAG)*
Questioning treatment utility	*Well I put drops in every night but I don’t actually know if they’re doing anything. L020 (Severe OAG)*
Factors influencing treatment burden	*Well, it’s different for me because I’m retired obviously, I’m 72. It doesn’t bother me that way, I mean your eyes go a bit watery and a bit blurred for about ten minutes but that doesn’t matter. I’ll just sit in the chair and wait till it’s cleared a bit and then go out. It really doesn’t bother me having to take them twice. L004 (Early OAG)*
Forming habits	*I can’t remember when I last forgot to take them but I have done it once or twice and, er, it’s just a habit, you know, the last thing before I go to bed. L010 (Early OAG)*
Longevity of treatment	*I’ve had the surgery, I don’t know how long this will last, that’s my question. Is this going to be there forever or will I have to be operated on, I don’t know? L011 (Early OAG)*
Life-long treatment	*It’s sort of been ingrained in me to just accept that I am most likely gonna be taking these drops for the rest of like, the drops at least, for the rest of my life. L019 (Early OAG)*
Treatment intensity	*Taking drops is, you do it at two times in a day twice, I have to take it in the morning and then before going to bed. So that is a problem where I can, I can sort it out by laser or any other way, it’s better. L007 (OHT)*
Treatment preferences	*I like to see, to be able to have one treatment, that solves the problem. L001 (Early OAG)*
Treatment stability	*The length of time I would like for it to stabilise that so much that I don’t have to do, to do anything more, erm, because I think, I don’t know what the next stage is. I’ve had the surgery, I don’t know how long this will last, that’s my question. L011 (Moderate OAG)*
Clinic attendance	*The only thing I didn’t used to like is the waiting time; that’s something you can’t help. L024 (Early OAG)*
Relationship with clinicians	*I was thinking of some of the conversations I’ve had the doctors whether I missed something, whether they were telling me, you know, do this, do that and I’ve missed it, but I don’t think so. I think they just said taking drops in morning and night and they didn’t, they didn’t treat my dry eyes. L004 (Early OAG)*
Success of treatment	*I’m using like you do the pressure number to tell me whether it’s successful. But really that just obviously doesn’t mean anything to me really in… at the end of the day. L014 (Severe OAG)*
**Theme 3. Most important outcomes to patients**	
Intraocular pressure control	*Definitely a reduced pressure, I want to see like a permanently reduced pressure. I get that it will fluctuate from time to time, but the idea is that it’s a reduced pressure. L019 (Early OAG)*
	*After the treatment the pressure dropped to 11. But today the pressure has gone back to 16. Though she said ‘It’s okay, still within the acceptable number’. So, I don’t think they should be using the pressure to gauge the success of the operation. L005 (OHT)*
Maintaining vision	*The outcome I want is obviously, success, but if it’s not successful, I do not want it to get worse, unless I have been informed that it could get worse. Then it’s my choice, I chose it, but I wasn’t expecting all this nonsense. L015 (Moderate OAG)*
Being independent	*I wouldn’t complain about it because I am not so practical about putting them in. If I had to, I would, but it’s so much easier for my wife to do it for me. L018 (Early OAG)*
Treatment that does not change	*The original thought… going back to last December was that I’d have one… you know… the Trabeculectomy… and then everything would be fine. L014 (Severe OAG)*
Freedom from eye drops	*I don’t like drops but you know I am pragmatic, so if I have to take drops I’ll take them, yeah. I am not, you know I just, I would look for a nice way to get away from them. If it’s possible through laser, erm you know I’d be happy as a sand boy. L018 (Early OAG)*
One-time treatment	*Well. it can be one time treatment that would really help my eye to see. L016 (Early OAG)*
**Theme 4. COVID-related concerns**	
Barrier to care	*On the one hand people are trying to keep you safe and on the other hand it’s been difficult to contact the doctor and to get follow up appointments and again, to find out whether you’re doing well or bad. L004 (Early OAG)*
Anxiety	*I was a bit worried because they were changing the appointment every time. L003 (Moderate OAG)*
Lifestyle changes	*There is always a conflict me and my employer. Because they think I’m taking a lot of break or they think I’m not looking at enough on the computer where I cannot be doing that because I need to look after my eyes. L008 (Severe OAG)*

Quotations are identified with a participant code and were chosen to be representative of the other quotations in each subtheme. Glaucoma severity is based on the worse eye.

Italic text denotes direct quotations from study participants.

**Fig. 3:**
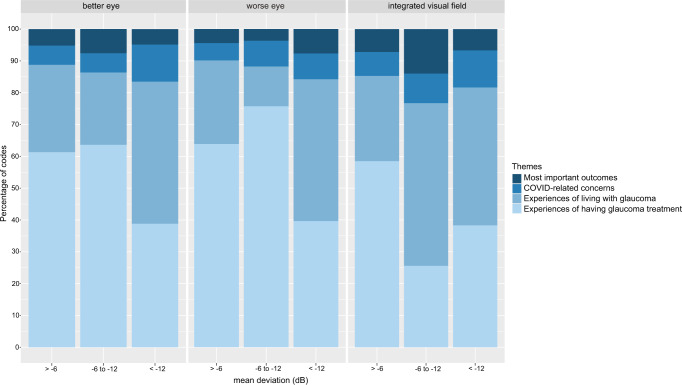
Percentage of total codes per theme categorized according to disease severity in worse eye or better eye or using integrated visual field. A larger area indicates a greater proportion of items coded.

To identify prominent themes, we analysed the proportion of coding references associated with each theme. Patients’ experiences of having glaucoma treatment (Theme 2) and patient’s experiences of living with glaucoma (Theme 1) both received the most extensive coverage in our interviews (Fig. 3). Of note, this coverage was consistently high across patients with mild, moderate and severe glaucoma, whether defined in terms of the better-seeing eye, the worse-seeing eye or binocular visual function.

### Theme 1 – Patients’ experiences of living with glaucoma

Living with OAG or OHT had far-reaching effects on patients’ lives. We identified 13 distinct subthemes. Limitation of activities was at the forefront of participants’ narratives. Routine, daily-life activities, such as walking, reading, and driving were disturbed, especially for participants with severe glaucoma. Some participants were worried about the effect of glaucoma on their careers. Limitation in performing specific vision-demanding activities led to a dependent life for some patients. Another patient described how these limitations impacted their self-confidence.

Participants discussed how the nature of glaucoma and its progression affected their lives. Fear of blindness was described by some participants, including some with early glaucoma. This interacted with the lack of symptoms in the early stages of glaucoma. For example, one participant explained that there were almost no obvious symptoms, which made her question the accuracy of the diagnosis. There was variability in how disease deterioration affected patients’ lives.

### Theme 2 – Patients’ experiences of having glaucoma treatment

Participants described how treatment with drops, laser, and surgery affected their daily life and productivity. Our analysis identified the most prevalent dimensions of treatment burden among participants.

A commonly reported consideration among patients with a history of surgical treatment was the postoperative period. One participant explained that she was worried that her operated eye would be affected because of the heat from cooking. Laser, on the other hand, was quick and convenient for some patients, with some reporting transient discomfort after the procedure. Some patients who had taken drops were concerned about drop-related adverse events and wanted to avoid them. They raised the issues of systemic effects such as overdosing and allergic reactions.

The perceived burden of treatment also included the task of understanding treatments and appraising them based on their intensity and stability. These factors interacted with each other. For example, patients often described having to learn names of drops, understand the medications used, and determine how long the treatment would last with sometimes limited information from clinicians. Lastly, attending multiple hospital appointments was also a problem in glaucoma care.

### Theme 3 – Most important outcomes to patients

Our analysis identified 6 distinct subthemes for the most important outcomes among participants. All quotations and themes emerged from asking patients directly about the most important outcomes following the treatments they had received throughout their care.

We categorized 3 of these subthemes (IOP control, maintaining vision, and being independent) as outcomes related to disease and 3 of these subthemes (treatment that does not change, freedom from eye drops, and one-time treatment) as outcomes related to treatment.

### Most important outcomes related to disease

#### IOP control

Many participants cited IOP control, though some participants did not associate it with noticeable vision changes. Contrary to the majority, some participants expressed the view that IOP control was not important (Table 2). Others reported that they did not understand how IOP may be linked to blindness.

#### Maintaining vision

Patients in our study cohort across a range of ages and disease stages expressed that maintaining current vision was of utmost importance. Specifically, participants whose vision had been severely affected by OAG were worried about deteriorating vision and were willing to consider any treatment to avoid losing their vision.

#### Being independent

The burden of visual loss among participants with severe OAG extended to inability to commute and other hallmarks of independence. Some participants with severe OAG relied on others, which made them feel dependent. Interestingly, participants with either early or severe OAG were concerned about the efforts they made to comply with medications. For instance, a participant with mild OAG explained that his wife always assisted him when instilling eye drops.

### Most important outcomes related to treatment

#### Treatment that does not change

Participants often had been using glaucoma drops for a long time with multiple changes.

#### Freedom from eye drops

Participants liked convenient and simple treatments, with freedom from drops being considered preferable. The amount of time and cognitive effort needed to maintain their treatment was also noted.

#### One-time treatment

Some participants did not consider ongoing treatment with eye drops as a success. One patient hoped that a new treatment would be discovered as a one-time treatment to stabilize her disease.

### Theme 4 – COVID-related concerns

This study was conducted during the COVID-19 pandemic in the UK. The limited mobility outside of the home led to feelings of worry, worsened by concerns about access to eye care services. Cancelled hospital appointments and limited access to pharmacy to get eye drops were a source of stress and anxiety.

## Discussion

This study explored outcomes that matter to patients diagnosed with glaucoma or OHT. To our knowledge, this is the first study to report patients’ preferences across a wide range of glaucoma severities and treatments. Our findings highlight how outcomes related both to disease and to treatment are important to patients. We planned from the outset to ask patients directly what they considered to be the most important outcomes. Using this method, we identified 6 outcomes of prime importance, of which 3 were disease-related and 3 were treatment-related.

This work builds on recent evidence about the burden of treatment in glaucoma and its negative impact on QoL [19]. We now demonstrate patients’ unequivocal interest in treatment-related outcomes, supporting the hypothesis that QoL in glaucoma may be influenced by the burden of its treatment.

The European Glaucoma Society Guidelines state that the goal of care for people with glaucoma is to promote their well-being and quality of life [25]. It is important to distinguish patients’ expectations from those of the clinicians who look after them because they may differ [26]. We were particularly careful to avoid bias from overlaying analysts’ personal opinions during scrutiny of transcripts by asking patients specifically what they considered to be the most important outcome. Only these items were included in Theme 3 (most important outcomes). For example, clinicians may prioritize slowing down disease progression [12] and patients did indeed show appreciation of disease stability. However, no patient actually identified it as the most important aim of glaucoma treatment from their perspective.

Interestingly, our study has shown that IOP is considered as the most important outcome by some patients. This is in keeping with previous work which ranked patient’s preferences [27, 28]. However, our work suggests that patients may also have different expectations to each other. The reasons for this need to be explored.

There is no clear consensus on whether the better-seeing eye or the worse-seeing eye has greater influence on vision-related quality of life [21]. Some investigators suggest that the MD of the better-seeing eye overestimates the impact of visual field loss and argue in favour of assessing binocular visual function [23, 29]. Others have reported the exact opposite [30]. Importantly, we found extensive coverage of both disease-related and treatment-related issues across the full range of glaucoma severity regardless of whether this was defined in terms of the better-seeing eye, worse-seeing eye, or binocular visual function. Therefore, our conclusion is unaffected by the ongoing debate about how to assess glaucoma severity.

This study was conducted in the midst of the COVID-19 pandemic. We observed that COVID-related concerns were mainly about limited access to eye care. Several participants expressed a fear of going to clinic. Yet, no participant identified these concerns as being of prime importance on direct questioning. We therefore think it is unlikely that patient expectations shifted due to the pandemic. Regardless, the presently identified themes are more likely to be of ongoing relevance than themes identified by investigators prior to the pandemic.

One of the strengths of this study is that it used purposive sampling of patients combined with a recruitment endpoint determined by saturation. These techniques together enabled us to generate rich responses from participants with diverse demographics, disease statuses and treatment histories [31]. For example, participants with OHT with a previous history of minimally invasive glaucoma surgery were recruited in our study. While this is not the commonest scenario for patients with OHT, we deliberately included such cases to ensure that everyone’s experiences and preferences were represented. Thus, our findings are comprehensive, not biased. By contrast, previous studies chose cohorts of patients with narrowly-defined disease severities and treatment histories [17, 18]. For example, Bicket et al. excluded the views of patients who had already experienced surgery [18]. In another study, the majority of recruits were Caucasians above 60 years of age [16], so it may not have captured adequately the views of younger patients and those of other races. The heterogeneity observed in our cohort allowed us to generate information-rich data and explore outcomes from a broad range of perspectives. For example, we found that some patients with early glaucoma were affected by fear of going blind, lending support to the idea that the mere diagnosis of a potentially blinding disease may impact quality of life in the absence of actual major vision loss [32].

We chose one-on-one interviews rather than focus groups for our study. Articulate, confident and motivated individuals contribute effectively to focus group discussions, but such studies may inadvertently exclude valid views of less articulate participants.

In terms of comorbidity, we ensured that participants’ views were not contaminated as result of non-glaucomatous ocular comorbidities. In one recent study, forty percent of patients had cataract in both eyes [17]. Since cataract is a cause of vision impairment distinct from glaucoma, identified themes of vision-dependent activities of daily living and problems with general visual function may have been contaminated by the influence of cataract. In our study, patients with non-glaucomatous causes of vision impairment were excluded to ensure that elicited preferences were related only to glaucoma.

### Limitations

We recognise some limitations to our study. We recruited from only two hospital centres in the UK, which may limit the extent to which our findings are generalisable. Nonetheless, we have ensured that a broad range of perspectives were covered by recruiting a diverse sample consisting of participants of various ages, genders, ethnicities, disease profiles and treatment histories, as demonstrated in Table 1. Moreover, by recruiting from two locations serving urban, suburban and rural populations, we aimed to include the full spectrum of social diversity across the UK. In common with previous qualitative studies, this permits a degree of conceptual transference in UK glaucoma patients under NHS care [33, 34].

Our findings are not statistically representative of the entire population. However, the aim of this study is to capture the range of patient preferences by maximizing variation in the sample. We ensured that the full diversity of attitudes was represented by continuing to recruit participants until thematic saturation was established. It is well recognized that thematic saturation can be reached with relatively modest numbers of participants [24]. In this study, saturation was reached following interviews with 25 patients. Whereas qualitative studies are ideal for in-depth exploration of participants’ attitudes, we acknowledge that it is not possible to draw quantitative inferences from this type of work. Whether preferences are stable with time for individual patients warrants further investigation.

## Conclusion

This study brings new perspectives on outcomes that are valued by patients who have glaucoma by directly exploring their experiences, future expectations and priorities. Glaucoma places unique burdens on patients, and we show that these can be related not only to the disease itself but also to its treatment. This needs to be considered in the further development of glaucoma PROMs if they are to measure quality of life successfully.

## Summary

### What was known before

- Preserving health-related quality of life (QoL) is the ultimate therapeutic goal in glaucoma management.
- Existing patient-reported outcome measures (PROMs) used to evaluate QoL in recent clinical trials have been criticised for being insufficiently sensitive to capture changes in health status.
- Current measures of QoL tend to emphasise the effects on the patient of the disease itself.

### What this study adds

- By directly exploring patients’ outcome preferences, we found that patients care not only about the disease itself but also about the burden of its treatment.
- Both treatment-related and disease-related outcomes may need to be assessed when evaluating quality of life in glaucoma.

## Data Availability

The data that support the findings of this study are not openly available to avoid compromising individual privacy. However anonymised data are available from the corresponding author upon reasonable request.
